# Learning analytics: Data sets on the academic record of accounting students in a Nigerian University

**DOI:** 10.1016/j.dib.2018.06.078

**Published:** 2018-06-26

**Authors:** Folashade O. Owolabi, Pelumi E. Oguntunde, Dorcas T. Adetula, Samuel A. Fakile

**Affiliations:** a Department of Accounting, Covenant University, Ota, Nigeria; b Department of Mathematics, Covenant University, Ota, Nigeria

**Keywords:** CGPA: Cumulative Grade Point Average, GPA: Grade Point Average, First Class: CGPA between 4.50 and 5.0, Second Class Upper Division: CGPA between 3.50 and 4.49, 100 level result: First year result, Final CGPA: CGPA at graduation, Academic performance, Accountants, Exploratory data analysis, University, Nigeria

## Abstract

This paper presents data on the academic performance of a particular set of accounting students from the year of inception into a Nigerian university to the year of graduation. Descriptive analysis was performed on the dataset and a regression model which is capable of making predictions was fitted to the dataset. From the dataset, 24 out of the students who started with a first class result (CGPA above 4.50) still maintained a first class result at graduation. 4 out of the students who started with a first class result dropped to second class upper division before graduation. 4 out of the students who started with a second class upper division result moved to first class result before graduation. 28 out of 35 students who started with a second class upper division maintained a second class upper division result at graduation.

**Specifications Table**

**Table:** 

Subject area	Education
More specific subject area	Accounting Education
Type of data	Table and text file
How data was acquired	Primary data
Data format	Raw, analyzed using descriptive and inferential statistics
Experimental factors	Data sets on the academic record of a set of Accounting students from their year of admission into the University to the year of graduation
Experimental features	Information on the grade point average (GPA) and the cumulative grade point average (CGPA) of a set of Accounting students between their first academic session to their last academic session on campus
Data accessibility	Dataset is available in this article

**Value of the data**

• The data can be useful in the field of Education, Guidance and Counseling, Psychology, etc.
• The data can be useful for decision makers especially in the Ministry of Education.
• The data can be used to make predictions of academic performance.
• Statistical tools like chi-square test of independent, correlation analysis, regression analysis, time series analysis and so on can be applied to the data to make informed decisions.

## 1 Data

The dataset used in this study represents the GPA and CGPA of some undergraduate students. The students studied accounting and their academic performance was monitored for their four years on campus. The dataset involves seven (7) missing data points; details can be viewed in Supplementary data. These missing data are highlighted in red and they represent information of (a) students who probably did not gain admission to study accounting but later changed to accounting in the second year following his/her outstanding performance in a closely related department in the first year (b) students who probably did not perform well or meet up with minimum promotion requirements and eventually dropped out from accounting department or entirely from the school (c) students who probably could not graduate with their mates s a result of failed courses, health issue and other personal reasons. The missing data points were eliminated totally for the sake of analysis. The dataset is summarized in Table 1.

**Table 1:** Summary statistics of the data.

	100 Level CGPA	Final CGPA
Minimum	2.1400	2.0262
Maximum	4.9500	4.9562
Mean	4.1290	4.0058
Variance	0.3554	0.4554
Skewness	−0.99	−0.61

**Fig. 1:**
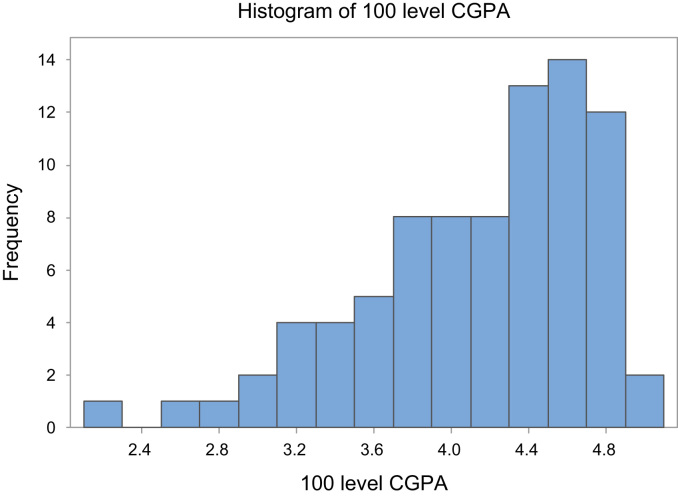
Graphical representation of the 100 level CGPA.

**Fig. 2:**
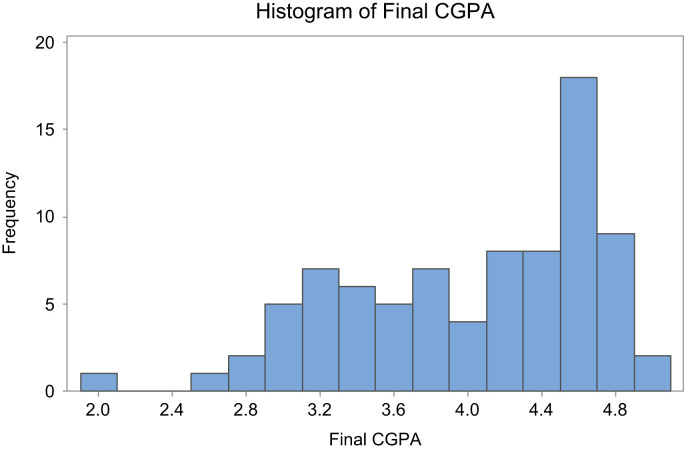
Graphical representation of the Final CGPA.

The dataset is represented graphically in Figs. 1 and 2.

## 2 Experimental design, materials and methods

Nigeria like other countries in the world has higher institutions of learning with reputes. These higher institutions are either privately owned (by individuals or religious institutions) or owned by the government. It is common in Nigeria that students would naturally want to study courses like Medicine, Engineering, Accounting and Computer Science (among others), hence the choice of Accounting for this study. Some recent studies on learning analytics include the works of Refs. [1], [2], [3], [4], [5] and the references therein.

**Fig. 3:**
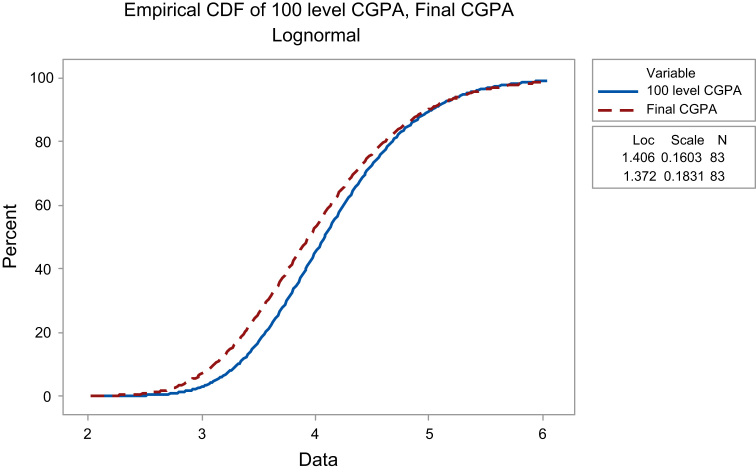
Comparing the empirical cdf of the datasets.

Since the dataset is skewed (as indicated in Table 1), we considered using Lognormal distribution in describing how far or close the 100 level CGPA is to the Final CGPA. Other standard distributions like Weibull distribution, Gamma distribution and so on can be used, we refer readers to Ref. [6] about these distributions. The result is presented graphically in Fig. 3.

### 2.1 Test for equality of means

The curves in Fig. 3 did not show too many dispersion, we further used t-test to know if there is a significant difference between the mean of the 100 level CGPA and the Final CGPA. The specific hypothesis used is:

Hypothesis I:

H_0_: There is no significant difference between the mean of the 100 level CGPA and the Final CGPA of the students

Versus

H_1_: There is a significant difference between the mean of the 100 level CGPA and the Final CGPA of the students

The null hypothesis is rejected if the p-value is less or equal to the level of significance (0.05). The result of the t-test is displayed in Table 2.

**Table 2:** Result of the *t*-test for equality of means.

Difference in the means	0.1232
95% Confidence Interval (C.I) for the difference	(−0.0720, 0.3184)
*T*-Value	1.25
*P*-Value	0.214
Degree of Freedom (DF)	161

The *p*-value in Table 2 (0.214) is greater than 0.05 (the level of significance), so, there is no enough evidence to reject the null Hypothesis. In other words, we accept the null hypothesis and conclude that there is no significant difference between the mean of the 100 level CGPA and the Final CGPA of the students.

### 2.2 Regression analysis

A simple linear regression model is fitted on the dataset to show the linear relationship between the Final CGPA and 100 level CGPA. The Final CGPA is taken to be the dependent variable while 100 level CGPA is the independent variable. The fitted regression line is:

(1) FinalCGPA=−0.237+1.0275(100levelCGPA)

For every unit increase in 100 level CGPA, there is a corresponding increase of 1.0275 increase in the Final CGPA of the students. Hence, the Final CGPA of a student can be predicted from the expression in Eq. (1). The summary of the model is presented in Table 3.

**Table 3:** The model summary.

R-square	R-square (adjusted)	R-square (predicted)
82.38%	82.16%	81.56%

The R-square value of 82.38% means about 82.38% of the variability in the Final CGPA is being explained by the 100 level CGPA.

A test for the significance of the regression model is conducted and presented in the Analysis of Variance (ANOVA) table in Table 4. The hypothesis used is:

**Table 4:** ANOVA table.

Source of Variation	DF	Sum of Square	Mean Square	*F*-value	*P*-value
Regression	1	30.764	30.7643	378.67	0.000
Error	81	6.581	0.0812		
Total	82	37.345			

Hypothesis II:

H_0_: The regression model does not significantly fit the data

Versus

H_1_: The regression model significantly fits the data

Since the *p*-value in Table 4 is 0.000 which is less than 0.05, then, we conclude that the regression model significantly fits the data. Also, the test for the significance of the regression parameter is conducted using *t*-statistic and the result is displayed in Table 5.

**Table 5:** Test for individual regression parameter.

Term	Coefficient	Standard Error of Coefficient	*T*-value	*P*-value
Constant	−0.237	0.220	−1.07	0.286
100 level CGPA	1.0275	0.0528	19.46	0.000

It has been confirmed in Table 5 that the variable we used (100 level CGPA) contributes significantly to the rejection of the null hypothesis.

**Important Points**

• CGPA does not significantly increase if the first year result is not good enough.
• There is a significant relationship between the Final CGPA and First year CGPA.
• From this research, there is no significant difference between the mean of the First year CGPA and the mean of the Final CGPA.
• Other methods which are available in Refs. [7], [8], [9] can be used to analyze the dataset to get other results.
